# An investigation of depression and inflammation as potential mediators linking adverse childhood experiences with cognitive decline in adulthood: results from a prospective cohort study

**DOI:** 10.1192/j.eurpsy.2022.546

**Published:** 2022-09-01

**Authors:** E. Lowry, A. Mc Inerney, N. Schmitz, S. Deschenes

**Affiliations:** 1 University College Dublin, Psychology, Belfield, Ireland; 2 University of Tübingen, Medicine, Tubingen, Germany

**Keywords:** adverse childhood experiences, inflammation, Depression, cognitive decline

## Abstract

**Introduction:**

Adverse childhood experiences (ACEs) have been associated with numerous health consequences in adulthood including cognitive decline. However, the underlying mechanisms implicated remain unclear.

**Objectives:**

In this study, depressive symptoms and systemic inflammation were investigated as potential independent mediators of the association between ACEs and cognitive decline.

**Methods:**

Participants were adults aged 50+ from the English Longitudinal Study of Ageing (N = 3,029; 54.8% female). Measures included self-reported ACEs at wave 3 (2006-2007), C-reactive protein (CRP) and depressive symptoms at wave 4 (2008-2009), and cognitive function at waves 3 and 7 (2014-2015). Mediation analyses examined the direct associations between ACEs and cognitive function at wave 7 and the indirect associations via depressive symptoms and CRP at wave 4 and were conducted using ordinary least squares regression models with the SPSS PROCESS macro. In Step 1, models were adjusted for sociodemographic factors and baseline cognitive function. Models in Step 2 were additionally adjusted for obesity and health behaviours (n = 1,874).

**Results:**

Cumulative ACEs exposure was shown to positively predict later-life depressive symptoms, which in turn predicted cognitive decline. ACEs were also shown to positively predict systemic inflammation as measured by CRP. However, CRP did not mediate the association between ACEs and cognitive decline.

**Conclusions:**

These findings suggest that ACEs are related to cognitive decline partly via depressive symptoms and corroborate prior research linking ACEs with adult systemic inflammation. Efforts towards screening for, preventing, and mitigating the effects of ACEs may therefore represent an important avenue for improving health outcomes in later life.

**Disclosure:**

No significant relationships.

